# The agricultural contamination elements (ACE) dataset: Multi class annotated images

**DOI:** 10.1016/j.dib.2026.112726

**Published:** 2026-04-03

**Authors:** Sean Donohoe, Femi Peter Alege, Christopher D. Delhom, William Blake Houston

**Affiliations:** a USDA-ARS, Cotton Ginning Research Unit, 111 Experiment Station Rd, Stoneville, MS 38776, USA; b USDA-ARS, Sustainable Water Management Research Unit, Stoneville, MS 38756, USA; c Texas A&M University, Biological and Agricultural Engineering Department, College Station, TX 77843, USA

**Keywords:** Machine vision, Precision agriculture, Cotton, Plastic, Aerial imaging, Benchmark

## Abstract

This work introduces the new agricultural contamination elements (ACE) dataset, which is comprised of annotated images representing four classes of elements, including bag, bottle, can, and trash. The bag annotation tag includes plastic bags and thin plastic sheet material. The trash annotation is a general-purpose tag for anything that does not fit into one of the other categories and that is not cotton. All the annotations included in the dataset are of the bounding box type. An unmanned aerial system (UAS) captured the images used for the annotations. The data capture included random heights and speeds to allow for more variation in the dataset. The current dataset includes images of cotton fields in Mississippi from three growing seasons (2021, 2022, and 2023). Researchers randomly placed the contamination elements in the cotton field before imaging and removed them from the field immediately after. The elements used within each class were random, based on what was available. The items serve as a general example of trash types and are not necessarily the most common examples of any type. In addition to three years, the data also represents different stages of the growing season. The data collected in 2021 contained the most variation in growing stages. The focus of the 2022 and 2023 data is defoliated cotton plants just before harvest. There are over 21,500 box annotations in the dataset, with 2021 accounting for 59%, 2022 accounting for 12% and 2023 accounting for 29%. The full-size images from the UAS were either still images taken at 16 megapixels (MP) or 4 K video. In either case, the full-size images were pre-processed and broken into square tiles of size 720×720 pixels with no overlap in height but some overlap in width. The extensible markup language (XML) files contain all the annotations and share the same name as their associated image. Folders separate the images and annotations by year to facilitate future studies that incorporate temporal aspects, such as testing performance across years.

Specifications Table

**Table:** 

Subject	Computer Sciences
Specific subject area	This work describes a dataset used in building machine vision systems.
Type of data	Images (.jpg format), Annotations in XML format
Data collection	The dataset includes annotated images of cotton fields at various growing stages of the crop season. The fields were prepared by randomly adding contamination prior to imaging. The collected data spans three crop seasons in Mississippi including: 2021, 2022, and 2023. A UAS captured the image data using both 16MP still images and 4 K (8MP) video. The data includes captures at random heights and random speeds, in addition to the various stages of growth. Researchers manually annotated the contamination elements after collection.
Data source location	The data came from fields around Stoneville, MS as part of a research project by the USDA-ARS Cotton Ginning Research Unit.
Data accessibility	Repository name: Annotated cotton field image dataset for contamination detection and removal Data identification number: https://doi.org/10.15482/USDA.ADC/30,138,301
Related research article	Donohoe, S.P., F.P. Alege, and C.D. Delhom (2026). Toward Multi-Class Detection and Localization of Contamination in Cotton Fields: Comparing EfficientDet, Faster R-CNN ResNet50, SSD MobileNet V2, and YOLOv11. Computers and Electronics in Agriculture, 247, 111690, https://doi.org/10.1016/j.compag.2026.111690.

## 1 Value of the Data

• This data is useful for developing computer vision systems for detecting and localizing multiple classes of contamination in cotton fields including plastic bags, bottles, cans, and general trash.
• A large number of annotated objects (over 21,500) provide examples of real-world fields for training robust agriculture focused vision systems especially in cotton fields both pre-and-post defoliation.
• The multi-year structure of the dataset also includes diversity in the cotton crop growth stages. This structure enables training models and testing their generalization over time.
• The annotated images include a high percentage of medium sized objects for benchmarking vision systems against challenging conditions.
• Agricultural engineers, computer vision experts, computer scientists, and others can use this data when researching, developing, or testing modern and classical computer vision systems.

## 2 Background

The dataset presented here was used to develop Artificial Intelligence (AI) based vision systems for detecting foreign matter in cotton fields [1]. Providing open access to the data used in the project will allow others to build upon those efforts. Plastic contamination in a bale of ginned cotton negatively impacts its economic value and leads to significant economic loss. It is estimated that the potential presence of plastic in US cotton has eroded the price premium and caused a $750 million decrease in the annual value of the crop [2]. Contaminants in the field prior to harvest are a potential source of plastic found in ginned cotton. Some previous research has focused on removing plastics at various stages of post-harvest processing [[3], [4], [5]]. A challenge of the post-harvest detection and removal approach is that harvesting and post-harvesting machinery have the potential to break the plastics into smaller pieces. The small pieces are more difficult to detect and remove. Other works have investigated the potential of detecting plastic in the field manually [6,7]. A recent work showed great potential to detect plastic bags in a mostly green cotton field using YoloV5 [8].

## 3 Data Description

**Fig. 1:**
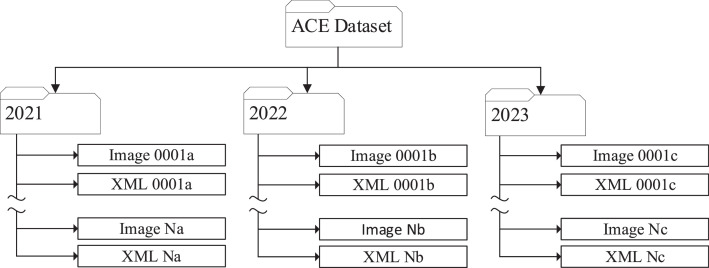
Dataset file structure.

The main directory comprises a series of folders that group the individual files by the crop year (2021–2023). Each named directory (2021–2023) contains a set of images in the joint photographic experts group (jpg) format and a set of extensible markup language (XML) files that describe the elements in the image. In the case of this dataset, the elements are all objects. The XML files are associated with the images through the file names, with the image and associated XML file having the same name except for the file extension. Fig. 1 illustrates this arrangement. The images are all 720×720 pixels with a square aspect ratio and are of the typical RGB format. The folder names represent the crop year of the data contained in them, and the folders contain different numbers of files depending on the number of annotated images for that year. The file names of the individual images and XML annotations are a mixture of alphanumeric characters that are unique across all the data. As such, it is safe to combine the data from multiple years into a single file if required.

The XML files contain a description of the elements or objects in the image. The XML files are designed to be compatible with the PASCAL Visual Object Classes (VOC) format [9]. Table 1 provides an example of a typical XML file. The VOC format includes several tags in the XML file that the ACE dataset does not currently utilize. The dataset includes these tags for compatibility reasons to keep the format like the original PASCAL VOC format, and for possible future use. These tags include “segmented”, “pose”, “truncated”, and “difficult”. The “segmented”, “truncated”, and “difficult” tags are all currently set to 0; the “pose” tag is currently always set to unspecified. In addition to the tags found in the original VOC format, the current dataset includes a GPS tag in the XML files. The XML file uses the GPS tag to store the GPS latitude and longitude that the camera system may have embedded in the EXIF of the source image. If no GPS data is available, then the tag displays a placeholder as “0.0, 0.0, 0.000”. The data in the dataset has had all GPS locations removed, however, the tag allows the inclusion of GPS data into future collected data as part of any dataset expansion. A discussion focusing on the use of the GPS tag is included in [1], such that the resultant XML file can be used to locate the objects detected.

**Table 1:** Example annotation formatting that matches Pascal VOC style [9] including key components.

<annotation>
<filename>image0001.jpg</filename>
<path>./2021_2022_2023/2021/image001.jpg<path>
<size>
<width>720</width>
<height>720</height>
</size>
<segmented>0</segmented>
<gps>
<value>
<lat>0.0 0.0 00.0000</lat>
<long>0.0 0.0 00.0000</long>
</value>
</gps>
<object>
<name>MY_OBJECT_NAME</name>
<pose>unspecified</pose>
<truncated>0</truncated>
<difficult>0</difficult>
<bndbox>
<xmin>MYVALUE</xmin>
<ymin>MYVALUE</ymin>
<xmax>MYVALUE</xmax>
<ymax>MYVALUE</ymax>
</bndbox>
</object>
</annotation>

There are four possible classes of contamination elements annotated in the dataset, including bag, bottle, can, and trash. The bag annotation tag includes plastic bags and thin plastic sheet materials. The bag class of elements is of particular importance in the dataset since wind can carry bags and transport them deeper in the field and they can become intertwined with the plants. The individual elements in this class are also sometimes located at different heights of the plant from the ground to the top of the canopy. As such the bag annotation may include more variation in the visual representation of the elements identified. The bottle and the can classes are both similar because their definitions are narrow, and they exist mainly on the ground level. The elements or objects within these classes can look similar as well since both are generally cylindrical and many are similar in scale. However, all the bottles were plastic while the cans were metal. The can definition includes only drinking type cans (soda, water, etc.) and does not include food cans. The trash class includes the food type cans. The trash class of the annotations is a general-purpose tag for anything that does not fit into one of the other classes. The elements in the trash class may contain the widest variation due to the relatively loose definition of the class. Some elements in this category may include food type cans, paper bags, food wrappers, stray car parts, bits of foil, and others. There is no limit to the number of elements or objects annotated in an image and contained in the XML file. Table 2 includes cropped images of some typical objects taken from source images that span the full range of classes (bag, bottle, can, and trash). The images were padded with a solid green buffer to enforce a square aspect ratio, ensuring no distortion when visualized in the table. These examples also show some of the objects partially occluded by the plants while others are in clear view. The objects also range in visual clarity with some being easier to identify than others.

**Table 2:** Examples of contamination elements or objects cropped from the image tiles using the dataset annotations.

**Fig. 2:**
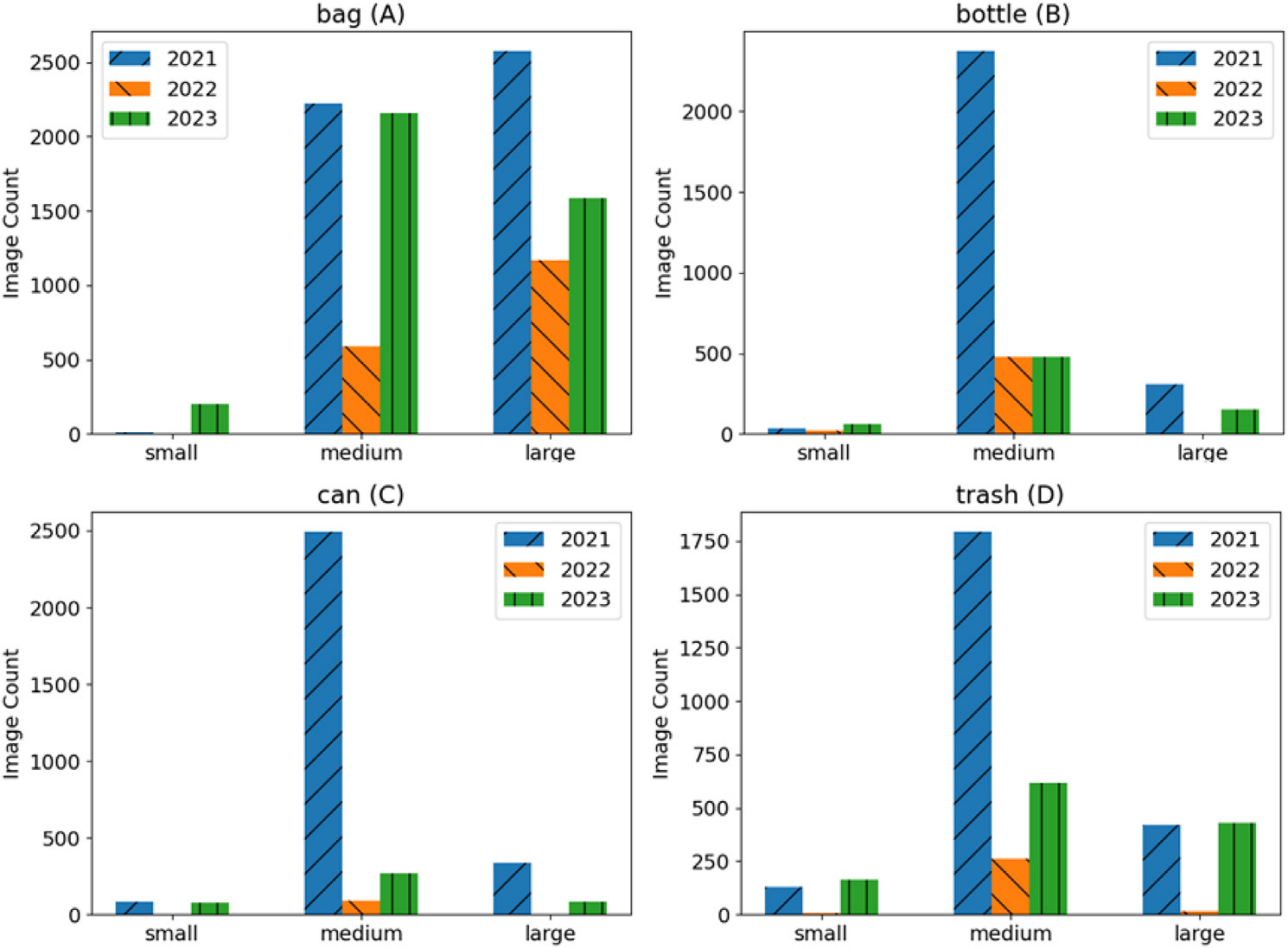
Dataset object size distribution by A) bag, B) bottle, C) can, D) trash.

There are over 21,500 annotated element objects in the dataset. Fig. 2 provides the size distribution of the objects in the dataset broken down by type. Fig. 2 left to right and top to bottom includes (A) bags, (B) bottles, (C) cans, and (D) trash. The objects within each class are further broken down by the crop year that they represent. To maintain compatibility with the Common Objects in Context (COCO) dataset in terms of the total pixels in an object, the size displayed on the horizontal axis of the plots are defined as “small”, “medium”, and “large” [10,11]. By that definition, small is less than or equal to 1024 pixels, medium is 1025 to <9216, and large is greater than or equal to 9216 pixels. Appendix A provides the code used to generate the statistics and plots presented using the dataset folder as an input. The object size of the elements in the bottle, can, and trash classes are primarily medium sized, with significantly fewer objects showing up in either the large or small categories. The object size distribution is different for the bag elements, with a relatively higher number of large objects in the data. Like the bottle, can, and trash objects (Fig. 2B-D), there are also a substantial number of medium-sized bag objects and very few small objects. The images from 2023 contain the largest number of small bag elements. With the differences in mind, these sizes do not represent the physical size of the objects but rather the apparent image size. For a given fixed lens camera, both flight height and camera angle affect the apparent image size. Overall, a large portion of the annotated elements or objects contain <9216 pixels.

**Fig. 3:**
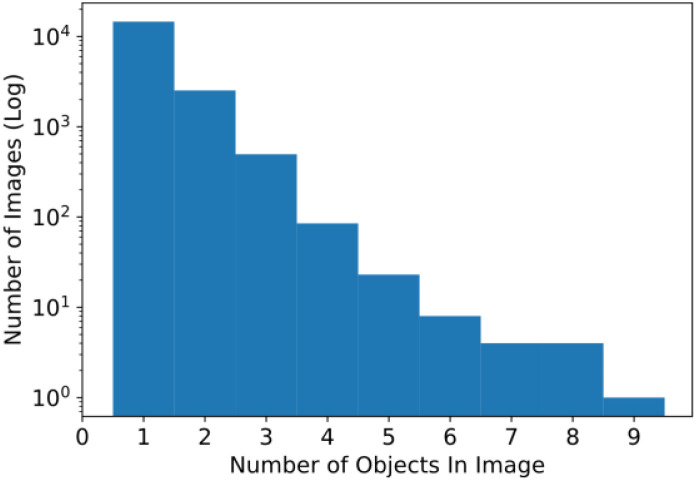
Histogram of the number of objects per image.

Fig. 3 provides a breakdown of the number of objects in each image across all years of the dataset. The scale of the y-axis is logarithmic so that the relatively low counts in some of the categories are still readable. The objects in each image range from one to nine, with most of the images having four or fewer.

## 4 Experimental Design, Materials and Methods

An unmanned aerial system (UAS) acquired the images while flying at random heights and speeds. The data included both still images and videos, with videos representing a large majority of the process. The camera orientation is typically facing straight down, perpendicular to the body of the UAS. However, some images included other camera angles. The dataset includes images captured by the UAS using both manual flights and automatically controlled flight paths. The arbitrary nature of the data collection exposes the training models to more real-world cases. As such, the method should help any model built using the data to generalize without needing strict requirements on the speeds or heights of the UAS platform. The UAS captured still images using a 16MP resolution (5472×3078 pixels) while the videos were in a 4 K format, which is just over 8MP resolution (3840×2160 pixels).

The dataset was constructed by imaging cotton fields that were prepared by adding contaminants prior to flying the UAS. Researchers placed items in the field indiscriminately from the ground to the top of the canopy, emphasizing the area of the field nearest to the road. For each data collection event, researchers placed contaminants arbitrarily in the preselected field. The exact items placed served as a general example of trash types and are not necessarily the most common examples of any type. It is very important to note that the presence or absence of an item does not imply that the exact item is either commonly found or never found on the roadside. The mix of items was occasionally changed with time. The collected data represents various stages of cotton production, including field preparation, cotton growth, and post-defoliation. Researchers removed the contamination after every imaging event and saved the materials for later reuse.

### 4.1 Image processing pipeline

As discussed in [1], the raw images captured by the UAS are too large for many object detection systems without applying drastic downscaling. As of 2025, the TensorFlow [12] model detection zoo has many models that require input image resolutions below 1280×1280 pixels, with some requiring much smaller sizes. Since the raw images from the UAS range from approximately 8MP to 16MP, these models could require a strong downscale which would have resulted in small-sized objects becoming proportionally smaller. With many annotated objects already under approximately 9000 pixels, strong downscaling would have shifted many objects into a smaller size category. Rather than downscale the images, the dataset used an alternate form of preprocessing. Tiling is a known preprocessing method for small object detection that can improve performance [13]. As such, the ACE dataset used image tiling to break the images into smaller pieces while preserving the small objects in the images.

**Fig. 4:**
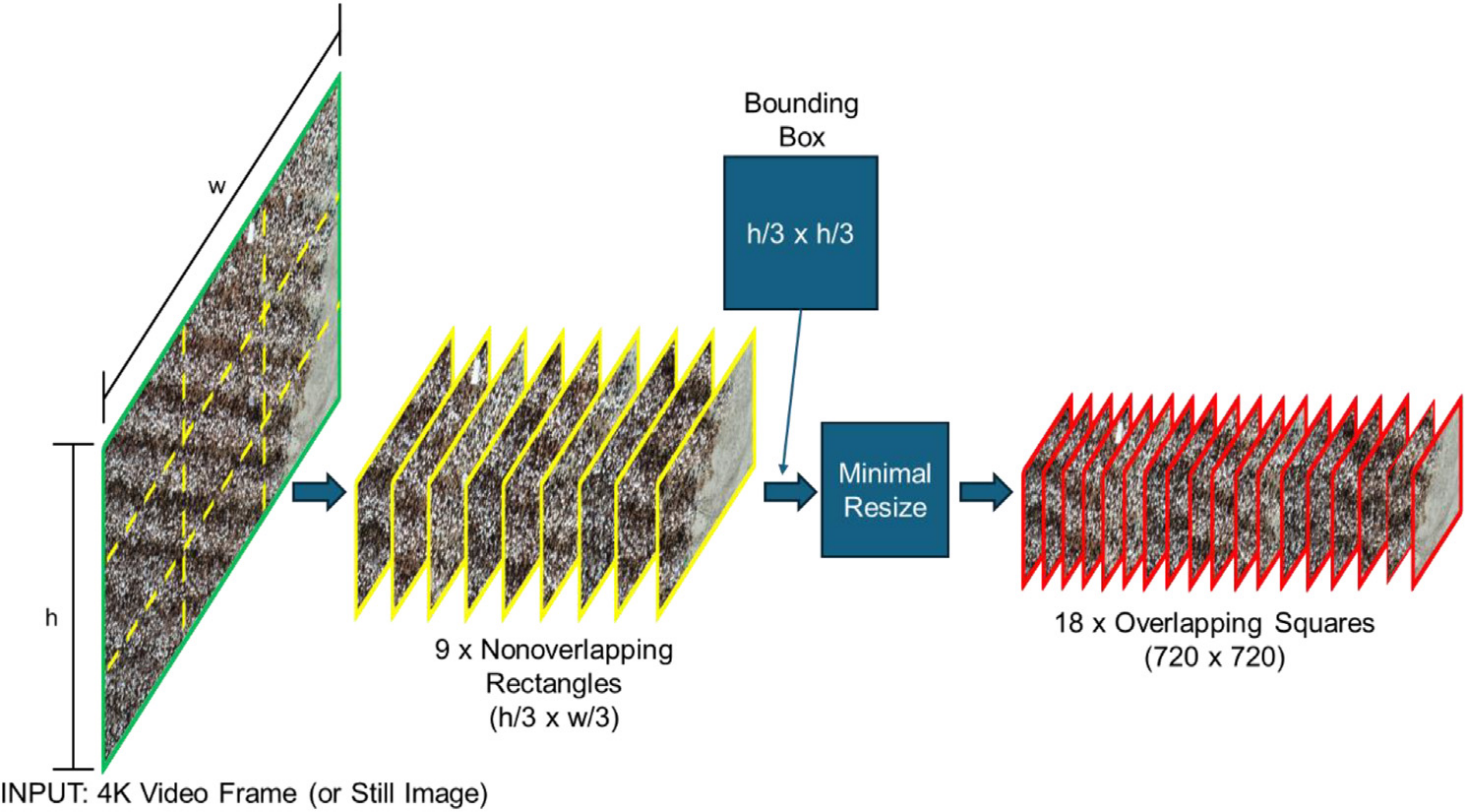
Decomposition of source image into square tiles.

The preprocessing portion of the code pipeline from Donohoe, Alege and Delhom [1] was used to tile the images in this dataset. Fig. 4 shows the steps involved in breaking the large source images into smaller tiles for annotation. This process can be broadly described to contain four steps: importing the full size image, breaking the image into nonoverlapping rectangles, breaking each rectangle into two overlapping squares, and resizing if needed for export. This process minimizes the amount of resizing needed and helps to preserve small objects in the images.

Elaborating on the individual steps of the preprocessing used, the first step was to load the source image and subdivide it into nxm rows and columns. For the purposes of this dataset, n=m=3. This resulted in nine rectangular images with no overlap each having a size of h/3xw/3 where h and w are the source image height and width respectively. When applied to a 4 K (2160P) source, equating n and m to three results in an image height of 720 pixels which was the final size.

**Fig. 5:**
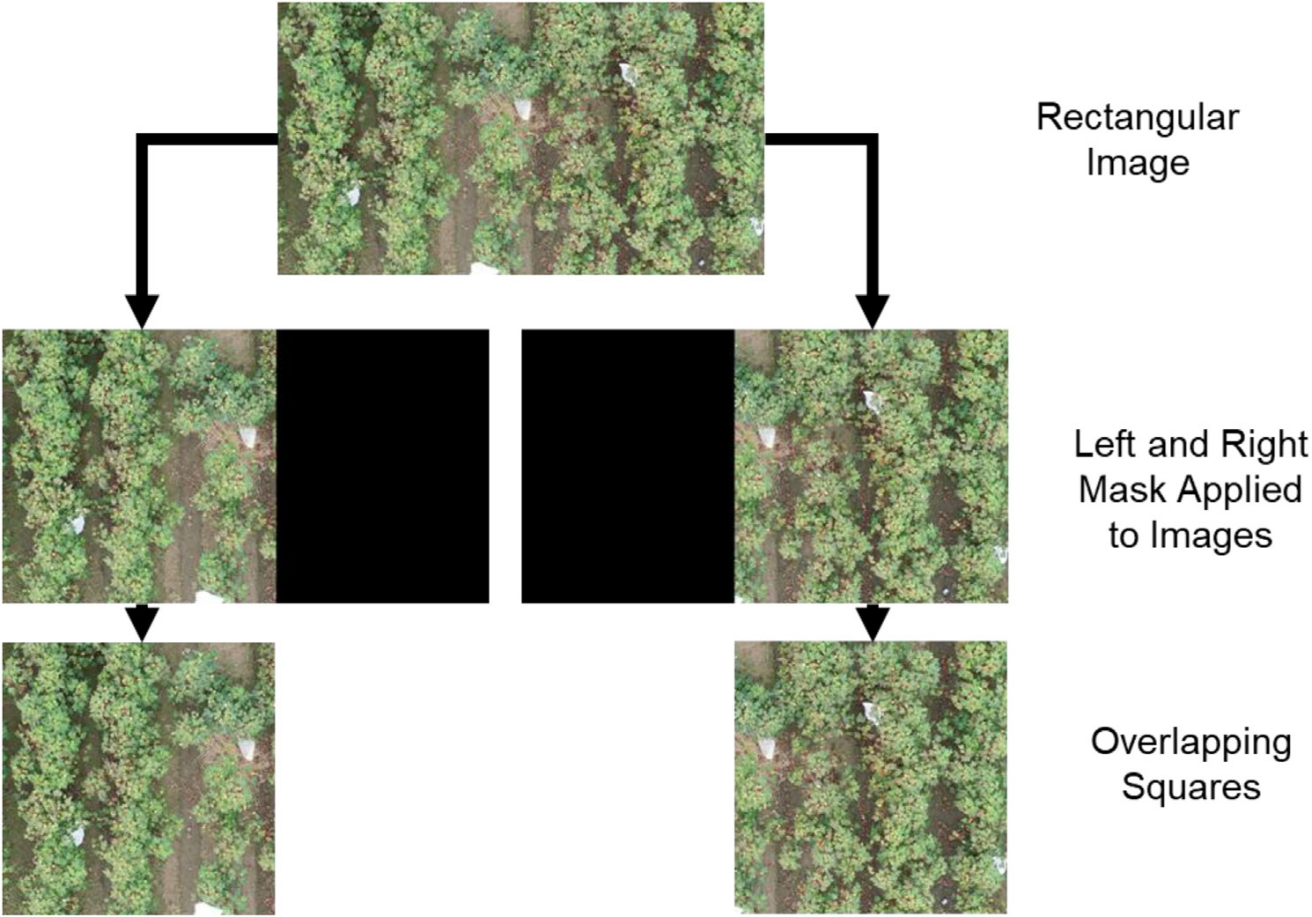
Decomposition of a rectangular image into two square images with some horizontal overlap.

In the next step, the software applied a bounding box that acted like a mask to each of the rectangular images. The bounding box was of shape h/3xh/3 and the software applied it twice to each rectangular image, resulting in two square images with some overlap. For this to work properly, the rectangular images could not have a width to height aspect ratio of >2:1. Fig. 5 shows a conceptual example of how the software applied the bounding box or mask to the nonoverlapping rectangular images to form two square images.

After the square images were generated, the software downsized them to 720 pixels square as needed. Since the square tiles are much smaller than the original source image, the magnitude of the resizing is minimized. In the dataset, the final square images have some overlap in the horizontal direction but none in the vertical direction. If images are added to the dataset using the same decomposition, the overlap will be related to the aspect ratio of the source image and the number of divisions used (rows and columns). If an image has an aspect ratio of 2:1 and n=m=3, each square image can have zero overlap. The minimal overlap helps by minimizing the total number of pixels that need to be inferenced when using the pipeline to detect objects. This process is unlike image stitching, where overlap is helpful. The overall process can preserve the Exchangeable Image File (EXIF) data in the original source image and copy the embedded values from the source to the final 18 overlapping squares.

With the video data, only every 4th frame was included in the dataset. Since the dataset was originally for an object detection project, every 4th frame represents a balance where each object includes a significant number of views while limiting the total number of annotations. More frames would likely be beneficial if image-based object tracking were desired.

### 4.2 Annotations and quality control

Multiple methods were used to generate the annotations in the dataset. These consisted of rectangular or square boxes manually drawn, boxes generated by AI and modified manually, and polygons manually drawn and converted into boxes. In all cases, the final dataset is comprised of box-type annotations. A quality control process checked all the final annotations as described later.

The first two annotation methods included manually drawn boxes and AI-generated boxes that researchers manually modified after generation. In the case of the AI-generated boxes, the AI assisted the manual process, and an operator manually corrected the outputs. Only the 2021 data used the AI assistant method. The AI assistant approach worked by training a model using a small subset of manually annotated data. The subset data AI model then pre-processed new images from the 2021 data. Researchers then manually reviewed the output, and corrected the predictions as needed. The corrections included removing false positives, filling in false negatives, modifying the detection classes, etc. This approach only applied to the 2021 data as a potential way to speed up the annotation process. The years 2022 and 2023 did not use this approach since initial testing did not save much time. The manual-drawn box method involved researchers viewing individual images, boxing objects, and identifying the type of object from one of the four classes. In both cases the annotations were of the box type.

Researchers manually annotated the 2023 data like much of the other data. However, rather than box annotations, these annotations were mainly of the polygon type. The polygons took significantly more time to complete but had the advantage of a finer resolution on the object while providing greater flexibility. For the purposes of this dataset, software converted the polygons into box annotations based on the maximum extents of the polygons.

**Fig. 6:**
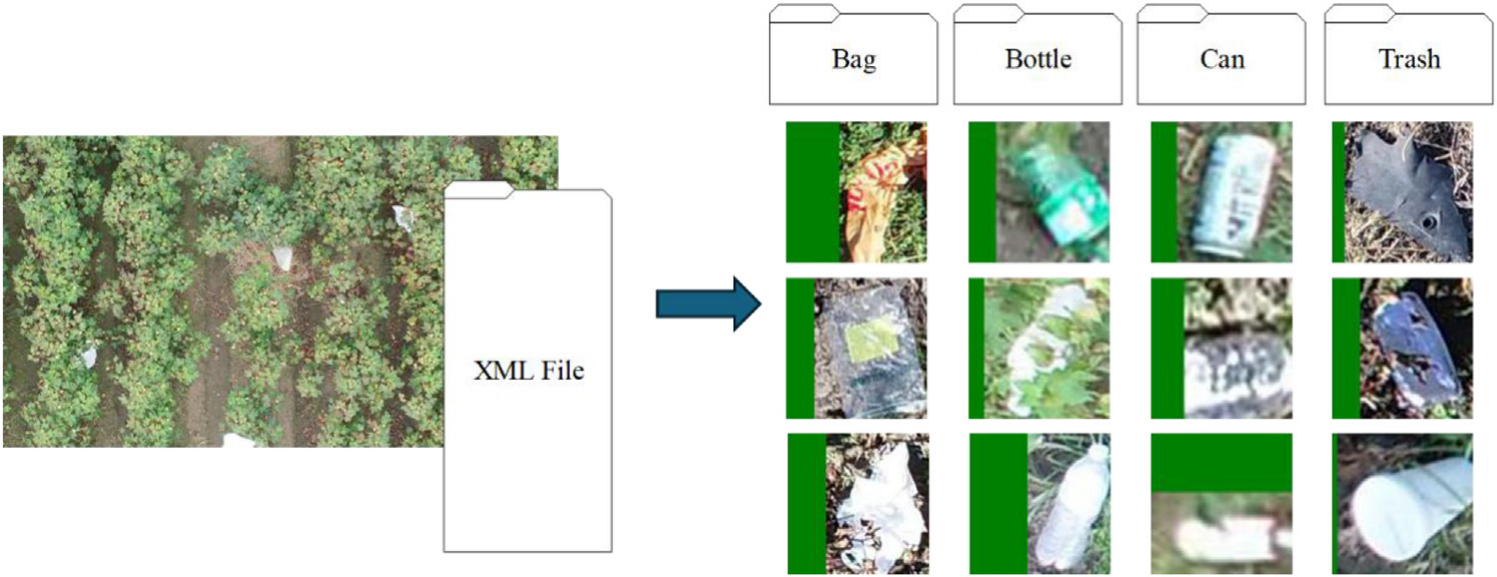
Example workflow for quality control of the data.

Quality control on the dataset included checks to ensure that annotations accurately identified objects in the images. Fig. 6 provides an example of how this process worked and Appendix B provides a minimal working example of the code written for this purpose. The first step in the process is fully automated and involves extracting and organizing the objects by type. The software did this by reading all the images and associated XML files from a user-specified folder. The XML files were then used to identify the bounding boxes of the objects and the type of objects contained in each image. The code then extracted all the objects and saved them as image patches. While extracting the objects, the software padded the image patch to preserve the aspect ratio of the objects. The code saved the image patches to folders that corresponded to the identified type of object the image patch represented. There was one folder for each type of bounding box (i.e., bag, bottle, can, and trash). The filenames of the image patches in each folder were generated by combining the source image name with a unique number ID.

The second step of the quality control process involved researchers manually reviewing each folder to confirm that every object in the folder was the correct type. Having folders that contain only a single class of elements allowed for viewing of multiple images simultaneously, which consumed less time than reviewing annotated images one at a time. If a researcher found any image out of place in a folder, they could use the name of the image to locate the source image and correct the annotation. This naming system allowed the researchers to map any errors detected back to the proper source image for addressing.

## Limitations

In this dataset the images represent a range of crop years, and a range of conditions (variable altitudes and speeds); the colors and patterns found on bottles, cans, and trash are not all-inclusive in this dataset due to the variety of materials available. The dataset was generated using RGB images and does not include multispectral or NIR which may limit its application for some types of segmentation.

## Ethics statement

The authors have read and followed the ethical requirements for publication in Data in Brief. Additionally, the authors confirm that the current work does not involve human subjects, animal experiments, or any data collected from social media platforms.

## CRediT author statement

**Sean Donohoe**: Conceptualization, Methodology, Software, Writing - Original Draft, Funding acquisition. **Femi Peter Alege**: Writing - Original Draft, Data Curation. **Christopher D. Delhom:** Writing -Review & Editing, Project administration, Funding acquisition. **William Blake Houston**: Writing - Original Draft, Data Curation.

## Disclaimer

Mention of trade names of commercial products in this publication is solely for the purposes of providing specific information and does not imply recommendation or endorsement by the U.S. Department of Agriculture. USDA is an equal opportunity provider and employer.

## Declaration of Competing Interest

The authors declare that they have no known competing financial interests or personal relationships that could have appeared to influence the work reported in this paper.

## Data Availability

The Agricultural Contamination Elements (ACE) Dataset: Multi Class Annotated Images (Original data).

## Appendix Supplementary materials

Image, application 1
