# Supplementary material for: The agricultural contamination elements (ACE) dataset: Multi class annotated images
Source: Data Brief. 2026 Apr 3;66:112726. doi: 10.1016/j.dib.2026.112726 (PMC13098605; doi:10.1016/j.dib.2026.112726)
Supplement: Supplementary file 1 [file mmc1.docx]

# APPENDIX A

Contains code used to generate the statistics of the data. Takes a directory and reads in all the folder contained in it. Generates the dataset statistics and plots used to describe the dataset.

import os

from os import path

import xml.etree.ElementTree as ET

import pandas as pd

import matplotlib.pyplot as plt

import numpy as np

class ReadXml:

'''

Class for reading XML files and getting bounding boxes

'''

def __init__(self, mypath): #path = the JPG file

self.xmi, self.xma, self.ymi, self.yma, self.object_l = ([],[],[],[],[])

self.xmlpath = mypath[0:len(mypath) - 3] + 'xml' #get name of xml file

self.total_pixels = []

self.stats_dict = {'small':0,'medium':0,'large':0}

def read_my_xml(self):

'''

Checks if XML file exists, read in all the objects, store in list.

'''

if path.exists(self.xmlpath):

root = ET.parse(self.xmlpath).getroot()

for t_obs in root.findall('object'):

self.object_l.append(t_obs.find('name').text)

self.xmi.append(int(t_obs.find('bndbox').find('xmin').text))

self.xma.append(int(t_obs.find('bndbox').find('xmax').text))

self.ymi.append(int(t_obs.find('bndbox').find('ymin').text))

self.yma.append(int(t_obs.find('bndbox').find('ymax').text))

#Add pixesl in box to list (can do stats on this later)

self.total_pixels.append((self.xma[-1]

-self.xmi[-1])*

(self.yma[-1]-

self.ymi[-1]))

#update the stats on the pixels

self.update_pixel_stats(self.total_pixels[-1])

else:

print('no file: ' + str(self.xmlpath))

def update_pixel_stats(self, size):

'''

Compare against small, medium, and large and update the dictonary

'''

if size<=1024:

#small

self.stats_dict['small']=self.stats_dict['small']+1

elif size<9216:

#medium

self.stats_dict['medium']=self.stats_dict['medium']+1

else:

#large

self.stats_dict['large']=self.stats_dict['large']+1

def get_total_boxes(self):

'''

Return number of boxes in XML

'''

return len(self.xmi)

def get_pixels_list(self):

'''

Return list showing number of pixesl in each box

'''

return self.total_pixels

def get_objects_list(self):

'''

Return the list of objects, if used with getPixelsList can

get dize distribution by types

'''

return self.object_l

def get_corners(self):

'''

Return type and (xmin, xmax, ymin, ymax)

'''

return (self.object_l.pop(0), self.xmi.pop(0), self.ymi.pop(0),

self.xma.pop(0), self.yma.pop(0))

def get_pixel_stats(self):

'''

Return dictonary showing the data about how many boxes in each

small medium and large category

'''

return self.stats_dict

def get_folder_names(directory):

'''

Get folders in the directory

'''

folder_names = []

for items in os.listdir(directory):

if os.path.isdir(os.path.join(directory, items)):

folder_names.append(items)

return folder_names

def get_items(input_folder):

'''

List of image names in the folder

'''

imageNameList = [input_folder+x for x in os.listdir(input_folder) if x[-1]=='g']

return imageNameList

def real_xml_vals(imagelist):

'''

Get values from the XML files return in DataFrame

'''

objectList, pixelList, numList = [],[],[]

smallList, medList, largeList = [],[],[]

for ii in imagelist:

val = ReadXml(ii)

val.read_my_xml()

objectList.append(val.get_objects_list())

pixelList.append(val.get_pixels_list())

numList.append(val.get_total_boxes())

px_sizes = val.get_pixel_stats()

smallList.append(px_sizes['small'])

medList.append(px_sizes['medium'])

largeList.append(px_sizes['large'])

#Convert to DataFrame

dfp = pd.DataFrame(pixelList, columns=max(numList)*['pixels'])

dfo = pd.DataFrame(objectList, columns=max(numList)*['objects'])

my_data = pd.DataFrame(imagelist, columns = ['name'])

my_data['Num'] = numList

my_data['small'] = smallList

my_data['medium'] = medList

my_data['large'] = largeList

return pd.concat([my_data, dfp, dfo], axis=1)

def total_by_type(mtpe, mydata):

'''

Tally object types

mtpe is a string specifying the type (i.e. "bag", "trash", etc.)

mydata is a data frame

'''

object_mask = mydata == mtpe #mask by object type (bool result)

object_mask = object_mask.rename(columns={'objects': mtpe+'objects'})

my_pixels = mydata.rename(columns={'pixels': mtpe+'objects'})

object_sizes = object_mask[mtpe+'objects']*my_pixels[mtpe+'objects']

total_objects = (object_sizes> 0).sum().sum()

return (total_objects, object_sizes)

def get_distribution(my_input):

'''

Get distribution of objects. my_input = dataframe with objects

'''

mask = (my_input > 0) & (my_input <= 1024) #small objects

small = mask.sum().sum()

mask2 = (my_input > 1024) & (my_input < 9216) #medium objects

medium = mask2.sum().sum()

large = (my_input>=9216).sum().sum()

return [small, medium, large]

INPUT_FOLDER = "./2021_2022_2023_CleanData/"

#get all the folders

myfolders = get_folder_names(INPUT_FOLDER)

#Creat dictonary of dataframes with the year as the key

my_dict = {}

for i, key in enumerate(myfolders):

myitems = get_items(INPUT_FOLDER+myfolders[i]+'/')

my_dict.setdefault(key, real_xml_vals(myitems))

#Setup fonts for plot

plt.rcParams['font.size'] = 14

plt.rcParams['font.serif'] = ['Times New Roman']

#Plot the size distributions by type

for item in ['bag','bottle','can','trash']:

TYPE = item

plt.figure()

categories = ['small', 'medium', 'large']

BAR_WIDTH = 0.2

x_pos_old = np.arange(len(categories))

patterns = [ "/" , "\\" , "|" , "-" , "+" , "x", "o", "O", ".", "*" ]

for i, key in enumerate(myfolders):

(objects, sizes) = total_by_type(TYPE, my_dict[key])

dist_list = get_distribution(sizes)

x_pos = x_pos_old + BAR_WIDTH

plt.bar(x_pos, dist_list, BAR_WIDTH, label=key, hatch=patterns[i])

x_pos_old = x_pos

plt.xticks(np.arange(len(categories)) + BAR_WIDTH*2, categories)

if item == 'bag':

IND = ' (A)'

if item == 'bottle':

IND = ' (B)'

if item == 'can':

IND = ' (C)'

if item == 'trash':

IND = ' (D)'

plt.title(TYPE + IND)

plt.ylabel("Image Count")

plt.tight_layout()

plt.legend()

#Plot the number of objects per image figures

total_list = []

for key in my_dict:

total_list = total_list + my_dict[key]['Num'].to_list()

plt.figure()

bin_edges = [0.5, 1.5, 2.5, 3.5, 4.5, 5.5, 6.5, 7.5, 8.5, 9.5]

plt.hist(total_list, bins=bin_edges)

plt.xticks(range(10))

plt.yscale('log')

plt.title('Objects Per Image')

plt.ylabel('Number of Images (Log)')

plt.xlabel('Number of Objects In Image')

plt.tight_layout()

#Total number of ojects

print('Total Objects: ' + str(sum(total_list)))

# APPENDIX B

Contains code used in the quality control routine by reading the XML values for each image in the given folder and cropping the objects from the image. Each object is placed into a folder representing the class of the object (or element).

from os import listdir, mkdir

from PIL import Image, ImageOps

import xml.etree.ElementTree as ET

from os import path

INPUT_FOLDER, OUTPUT_FOLDER = ("./check", "./cropsA")

IM_SIZE = 320 #size of output image (square with this dimension)

CLASSES = ['/can', '/bag', '/trash', '/bottle']

class readXML:

#Class for reading XML files and getting bounding boxes

def __init__(self, path): #path = the JPG file

self.xmi, self.xma, self.ymi, self.yma, self.objectL = ([],[],[],[],[])

self.xmlpath = path[0:len(path) - 3] + 'xml' #get name of xml file

def readMyXML(self):

#Checks if XML file exists, read in all the objects, store in list.

if path.exists(self.xmlpath):

root = ET.parse(self.xmlpath).getroot()

for objects in root.findall('object'):

self.objectL.append(objects.find('name').text)

self.xmi.append(int(objects.find('bndbox').find('xmin').text))

self.xma.append(int(objects.find('bndbox').find('xmax').text))

self.ymi.append(int(objects.find('bndbox').find('ymin').text))

self.yma.append(int(objects.find('bndbox').find('ymax').text))

else:

print('no file: ' + str(self.xmlpath))

def getTotalBoxes(self):

# Return number of boxes in XML

return len(self.xmi)

def getCorners(self):

# Return type and (xmin, xmax, ymin, ymax)

return (self.objectL.pop(0), self.xmi.pop(0), self.ymi.pop(0),

self.xma.pop(0), self.yma.pop(0))

#Lists to store info

image_list = []

image_name_list = []

#Make folder to store outputs

for c in CLASSES:

try: mkdir(OUTPUT_FOLDER+c)

except OSError as error: print(error)

#Add files from input folder into list

for f in listdir(INPUT_FOLDER):

if(f[-1] != 'l' and f[-2] != 'm' and f[-3] != 'x'):

image_list.append(INPUT_FOLDER+'/'+f)

image_name_list.append(f)

for i, name in enumerate(image_name_list):

mypath = image_list[i]

myreader = readXML(mypath)

myreader.readMyXML() #Read xml using path

TOTAL_BOXES = myreader.getTotalBoxes()

myI = Image.open(mypath)

for boxNum in range(TOTAL_BOXES):

#Get bounds of box and extract patch

bxtype, xminE, yminE, xmaxE, ymaxE = myreader.getCorners()

pat = myI.crop((xminE, yminE, xmaxE, ymaxE))

#pad image to square

width, height = (xmaxE-xminE, ymaxE-yminE)

if width>height:

tA = width-height

iPatch = ImageOps.expand(pat, border = (0, tA, 0, 0), fill="white")

if height>width:

tA = height-width

iPatch = ImageOps.expand(pat, border = (tA, 0, 0, 0), fill="white")

#resize, generate unique name based on number of images, and save

sized_patch = iPatch.resize((IM_SIZE, IM_SIZE), resample = 3)

num_files = len(listdir(OUTPUT_FOLDER+'/'+bxtype))

name = name.split('.jpg')[0]+'_'+str(num_files+1)

sized_patch.save(OUTPUT_FOLDER+'/'+bxtype+'/'+str(name)+'.jpg')
